# Cluster de Inatividade Física e Outros Fatores de Risco na Diabesidade em Adultos Quilombolas

**DOI:** 10.36660/abc.20230715

**Published:** 2024-11-13

**Authors:** Poliana Pereira Santana, Clarice Alves dos Santos, Ricardo Franklin de Freitas Mussi, Hector Luiz Rodrigues Munaro, Saulo Vasconcelos Rocha

**Affiliations:** 1 Universidade Estadual do Sudoeste da Bahia Jequié BA Brasil Universidade Estadual do Sudoeste da Bahia, Programa de Pós-Graduação em Educação Física UESB/UESC, Jequié, BA – Brasil; 2 Universidade Estadual do Sudoeste da Bahia Departamento de Ciências Biológicas Jequié BA Brasil Universidade Estadual do Sudoeste da Bahia, Departamento de Ciências Biológicas, Jequié, BA – Brasil; 3 Universidade do Estado da Bahia Caitité BA Brasil Universidade do Estado da Bahia, Programa de Pós-Graduação em Ensino, Linguagem e Sociedade, Caitité, BA – Brasil; 4 Universidade Estadual do Sudoeste da Bahia Departamento de Saúde II Jequié BA Brasil Universidade Estadual do Sudoeste da Bahia, Departamento de Saúde II, Jequié, BA – Brasil; 5 Universidade Estadual do Sudoeste da Bahia Departamento de Saúde Jequié BA Brasil Universidade Estadual do Sudoeste da Bahia, Departamento de Saúde, Jequié, BA – Brasil; 6 Universidade Estadual de Feira de Santana Feira de Santana BA Brasil Universidade Estadual de Feira de Santana, Programa de Pós-Graduação em Saúde Coletiva, Feira de Santana, BA – Brasil

**Keywords:** Assunção de Riscos, Diabetes Mellitus, Obesidade, Quilombolas

## Abstract

**Fundamento:**

A diabesidade é uma condição caracterizada pela coexistência de diabetes tipo 02 e obesidade. As causas são multifatoriais, resultantes de uma complexa interação de fatores genéticos e comportamentais. Entre os fatores comportamentais, destacam-se a inatividade física, os hábitos alimentares inadequados e o consumo excessivo de álcool e tabaco.

**Objetivo:**

Investigar o agrupamento (*clustering*) da inatividade física e outros fatores de risco e a associação entre as combinações de fatores de risco e a presença de diabesidade em adultos quilombolas.

**Métodos:**

Trata-se de estudo transversal com amostra composta por 332 adultos de meia idade e idosos (idade ≥ 50 anos), selecionados entre os participantes do estudo "Perfil epidemiológico dos quilombolas baianos". Os dados foram obtidos por meio de entrevistas e avaliação antropométrica. Para a análise dos dados, foram utilizadas estatísticas descritivas, análise de *cluster* e procedimentos de regressão logística multinominal.

**Resultado:**

A maior prevalência de agrupamento foi identificada para as combinações de consumo regular de álcool sem a presença dos demais fatores (O/E=14,2; IC95%= 0,87-1,15), seguido de consumo regular de álcool e tabaco (O/E=10,3; IC95%= 0,64-0,95) e consumo regular de álcool, tabaco e alimentos ricos em açúcar e gorduras (O/E=6,8; IC95%= 1,31-1,75). Na análise bruta, foram observadas associações entre inatividade física sem a presença dos demais fatores (OR= 0,82 IC95%= 0,78-0,86) e diabesidade.

**Conclusão:**

O consumo de álcool foi o fator mais prevalente nas maiores combinações avaliadas. Além disso, inatividade física, sem os outros comportamentos analisados, e a ausência de todos os comportamentos associaram-se à diabesidade apenas na análise bruta.

**Figure:**
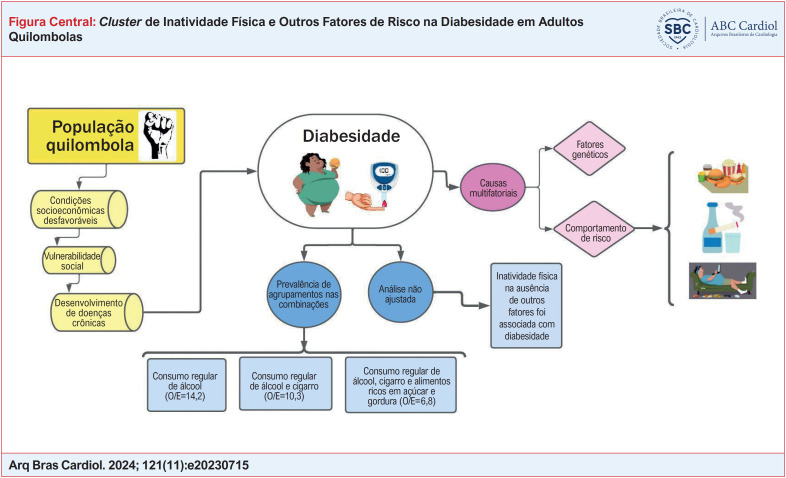


## Introdução

A Diabesidade é um termo usado para descrever efeitos adversos à saúde causados pela presença simultânea de duas condições – a obesidade e o diabetes mellitus tipo 2.^1^

A obesidade é um fator de risco importante para o desenvolvimento de diabetes tipo 2, ao desencadear resistência à insulina e complicações metabólicas.^2,3^ Estima-se que 80-90% dos indivíduos com diabetes tipo 2 são obesos e o risco está diretamente relacionado ao índice de massa corporal.^4^ Projeções indicam que, até 2025, mais de 300 milhões de pessoas desenvolverão diabetes mellitus tipo 2 associado à obesidade.^5^

As causas de obesidade e diabetes são multifatoriais e resultantes de uma interação complexa entre fatores genéticos e comportamentais. Fatores comportamentais importantes incluem hábitos alimentares inadequados, consumo excessivo de álcool e tabaco, e inatividade física. ^6,7^

A inatividade física é um sério problema de saúde pública e é responsável por mais de três milhões de mortes por ano em todo o mundo. Apesar disso, estimativas indicam que um terço da população mundial com idade acima de 15 anos não alcança a recomendação mínima da Organização Mundial de Saúde (OMS), isto é, a de se realizar no mínimo 150 minutos de atividade física por semana.^8^

Além da inatividade física, hábitos inadequados de alimentação parecem influenciar diretamente o desenvolvimento de obesidade e diabetes mellitus tipo 2. O consumo aumentado de carboidratos processados, com alto índice glicêmico, pode levar a alterações hormonais que promovem o acúmulo de tecido adiposo, exacerbam a fome, e reduzem o gasto energético.^9^ Por outro lado, a OMS recomenda o consumo de frutas e vegetais para reduzir a incidência de doenças cardiovasculares e certos tipos de câncer, bem como para prevenir e tratar o sobrepeso e o diabetes.^10^

Outro comportamento de risco que merece atenção é o tabagismo. Evidências indicam uma associação significativa entre tabagismo e uma prevalência mais alta de sobrepeso e obesidade, uma vez que os efeitos da nicotina favorecem o acúmulo de gordura abdominal.^11^ Ainda, o tabagismo promove importantes alterações metabólicas envolvidas no desenvolvimento de diabetes tipo 2. Entre elas, mostrou-se que a nicotina reduz a secreção de insulina, aumenta os níveis de cortisol plasmático, e induz disfunção e apoptose celular.^12^

Em relação ao consumo abusivo de álcool, estudos mostraram que o diabetes tipo 2 é causado por uma ação direta do álcool^13^ ou por outros comportamentos não saudáveis observados nos etilistas.^6^

Segundo a literatura, o processo saúde-doença é o resultado das condições de vida dos indivíduos, isto é, seus hábitos e estilo de vida, condições socioeconômicas, cultura e ambiente em que vivem.^14^ A partir dessa perspectiva, é evidente que condições socioeconômicas desfavoráveis aumentem a susceptibilidade a comportamentos de risco^15^ e ao desenvolvimento de doenças crônicas.^16^

Neste contexto, estudos brasileiros sobre desigualdades sociais que abordam raça/cor da pele demonstraram desfechos de saúde ruins na população negra.^17,18^

Entre os diferentes perfis das populações negras no Brasil, a população quilombola enfrenta uma situação de vulnerabilidade social,^18^ dada à dificuldade de acesso a políticas públicas e condições socioeconômicas inadequadas.^19,20^

Além disso, o diabetes sabidamente afeta as populações negras de maneira diferente, por influência dos fatores comportamentais, ambientais e genéticos, bem como pela vulnerabilidade socioeconômica.^21,22^ Um cenário similar parece se aplicar à obesidade.

Assim, condições econômicas podem influenciar negativamente a qualidade de vida de adultos e idosos quilombolas, que vivem condições desfavoráveis ao envelhecimento saudável.^23^ Estudos prévios investigaram comportamentos de risco e condições individuais de diabetes^6^ e obesidade^24^ em adultos e idosos. Os resultados indicaram que essas condições tendem a ocorrer simultaneamente, um fato que tem implicações de curto, médio e longo prazo. Contudo, estudos analisando combinações de fatores de risco relacionados à diabesidade ainda são incipientes, especialmente em idosos da população quilombola.

Nesse contexto, o objetivo do presente estudo foi investigar inatividade física e outros fatores de risco, e a associação entre combinações de fatores de risco e a presença de diabesidade em adultos quilombolas.

## Métodos

Foi conduzido um estudo populacional, transversal, descritivo, envolvendo uma população de remanescentes quilombola de uma microrregião de Guanambi, Bahia, Brasil, entre abril a novembro de 2016. A região é composta de 18 municípios e, durante o período de amostragem, havia 42 quilombos certificados, distribuídos em 10 desses municípios.^25^

O tamanho amostral foi calculado assumindo uma prevalência de desfecho desconhecido de 50%, intervalo de confiança de 95%, erro de amostragem de 5%. Ainda, foram adicionados tamanho do efeito de 1,5 para amostragem em uma fase de agrupamento, 30% de recusas e 20% para perdas de fatores de confusão. Outros detalhes do processo de seleção da amostra foram publicados anteriormente.^26^ Para o presente estudo, dados de adultos de meia idade e idosos (≥ 50 anos) foram incluídos, e a amostra consistiu de 348 indivíduos (40,8% da população total).

Os dados foram coletados por entrevista e medidas antropométricas, as quais foram conduzidas por equipes de profissionais da saúde e/ou estudantes de acordo com suas qualificações, após haverem sido treinados em suas funções. Os seguintes dados foram coletados: sexo (masculino ou feminino); idade em anos completos e categorizada por grupo etário (50-74 anos ou ≥ 75 anos); ocupação (trabalho remunerado ou não); estado civil – com ou sem parceiro(a); nível educacional (≤ 5 ou > 5 anos de escolaridade); tabagismo (sim: fuma ou já fumou; não: nunca fumou); consumo regular de álcool (sim: consome ou consome esporadicamente; não: nunca consome); consumo regular de frutas e verduras (sim: sempre, quase sempre, às vezes, não: nunca); consumo de alimentos ricos em açúcar e gordura (sim: sempre, quase sempre, às vezes, não: nunca); presença de diabetes (sim ou não).

Atividade física foi avaliada usando a versão curta do Questionário Internacional de Atividade Física (IPAQ, do inglês *International Physical Activity Questionnaire*),^27^ que acessa o tempo semanal gasto em atividade física moderada ou vigorosa em diferentes domínios da vida (trabalho, tarefas domésticas, transporte e lazer). Os indivíduos cuja soma de atividade física modera ou vigorosa nos diferentes domínios tenha sido menor que 150 minutos por semana foram classificados como insuficientemente ativos.

Diabesidade foi classificada com base no diagnóstico autorrelatado de diabetes mellitus e a medida da circunferência da cintura.^28^ A presença de diabesidade foi definida quando os participantes com um diagnóstico autorrelatado de diabetes apresentavam, simultaneamente, uma circunferência da cintura > 90 cm para homens e > 80 cm para mulheres.^29^

Estatística descritiva (frequências simples e relativa e medidas de dispersão) foi usada para análise univariada dos dados. Para análise bivariada, foi aplicado o teste do qui-quadrado de Pearson para comparar as variáveis entre homens e mulheres usando o *Statistical Package for the Social Sciences* (SPSS versão 22 para Windows).

A presença simultânea ou o agrupamento de fatores relacionados à diabesidade foi analisado pelo cálculo da probabilidade conjunta dos comportamentos exibidos. A presença de agrupamento foi avaliada comparando-se a prevalência Observada (O) e a prevalência Esperada (E). Agrupamento é definido quando a razão O/E for > 1. Para análise da associação entre os preditores com a diabesidade, estimou-se o Odds Ratio (OR) do modelo de regressão logística binária.

O estudo "Perfil epidemiológico dos Quilombolas na Bahia" foi aprovado pelo Comitê de Ética da Universidade Estadual do Sudoeste da Bahia (Opinião No. 1.386.019/2016) e conduzido segundo as diretrizes brasileiras sobre pesquisa envolvendo humanos, de acordo com a Resolução 466/2012 do Conselho Nacional de Saúde.

## Resultados

A idade média dos indivíduos incluídos no estudo foi 61,43 ± 9,46 anos, e a maioria dos indivíduos era do sexo feminino (52,5%). A prevalência de inatividade física, ausência de consumo de frutas e vegetais, consumo de bebidas alcoólicas, tabagismo, e consumo de alimentos ricos em açúcar e gorduras foi 23,6%, 41,8%, 25,6%, 47,9% e 89,9%, respectivamente. Além disso, 17,8% dos respondentes apresentaram diabesidade (Tabela 1).

**Tabela 1:** Associação entre características sociodemográficas e fatores de risco relacionados ao estilo de vida em quilombolas adultos vivendo no município de Guanambi, Bahia, Brasil

Variáveis		Amostra Total		Inatividade física		Falta de consumo de frutas e verduras		Consumo de bebida alcoólica		Tabagismo		Consumo de alimentos ricos em açúcar/ gordura	
		n	%	n	%	n	%	n	%	n	%	n	%
**Total**		320		61	23,6	132	41,8	82	25,6	148	47,9	284	89,9
**Sexo**				p=0,59		p=0,26		p=0,00		p=0,00		p=0,03	
	Masculino	153	47,8	31	20,3	68	44,4	68	44,4	103	67,3	130	84,9
	Feminino	167	52,5	30	17,9	64	38,3	14	8,4	45	26,9	154	92,2
**Grupo etário (anos)**				p=0,38		p=0,39		p=0,70		p=0,26		p=0,52	
	50 – 74	277	86,6	52	18,7	117	42,2	72	26,0	124	44,8	246	88,8
	75 ou mais	43	13,4	9	20,9	15	34,9	10	23,2	24	55,8	38	88,4
**Ocupação**				p=0,32		p=0,29		p=0,08		p=0,33		p=0,16	
	Trabalho remunerado	274	85,6	51	18,6	116	42,3	75	27,4	129	47,1	240	87,5
	Trabalho não remunerado	46	14,4	10	21,7	16	34,7	7	15,2	19	41,3	44	95,6
**Estado civil**				p=0,65		p=0,54		p=0,61		p=0,33		p=0,36	
	Com um(a) parceiro(a)	259	80,9	48	18,5	109	42,1	69	26,6	124	47,8	232	89,6
	Sem um(a) parceiro(a)	60	18,8	13	21,6	23	38,3	13	21,6	24	40,0	52	86,6
**Escolaridade**				p=0,001		p=0,07		p=0,66		p=0,86		p=0,71	
	≤ 5 anos	242	75,6	37	15,3	108	44,6	65	26,8	113	46,7	213	88,0
	> 5 anos	47	14,7	13	27,6	15	31,9	10	21,3	22	46,8	43	91,5
**Diabesidade**				p=0,46		p=0,72		p=0,12		p=0,91		p=0,70	
	Sim	57	82,2	12	21,0	25	43,8	10	17,5	26	45,6	52	91,3
	Não	263	17,8	31	54,3	32	56,2	47	82,5	29	50,9	5	8,7

Os resultados da Tabela 2 mostram a ausência de associações entre fatores de risco individuais e presença de diabesidade.

**Tabela 2:** Associação entre fatores de risco relacionados ao estilo de vida e presença de diabesidade

Fatores de risco	OR (IC95%)	OR ajustado (IC95%)
Falta de consumo de frutas e verduras	1,10 (0,62-1,98)	0,80 (0,40-1,59)
Consumo regular de bebida alcoólica	1,21 (0,44-3,29)	0,90 (0,28-2,89)
Tabagismo	0,97 (0,54-1,73)	0,72 (0,34-1,55)
Consumo de alimentos ricos em açúcar	0,56 (0,27-1,17)	1,32 (0,55-3,20)
Inatividade física	1,31 (0,63-2,76)	0,60 (0,27-1,31)

Ajustado por sexo, idade, escolaridade, estado civil, e ocupação. OR: Odds Ratio; IC: Intervalo de Confiança.

Entre as 32 possíveis combinações, escores de agrupamento (*clusters*) foram obtidos para seis combinações. Os escores mais altos foram identificados para as combinações de consumo regular de bebida alcoólica na ausência de outros fatores (O/E=14,2), seguido de consumo regular de bebida alcoólica e tabagismo (O/E=10,3), e consumo regular de bebida alcoólica, tabagismo e consumo de alimentos ricos em gordura e açúcar (O/E=6,8) (Tabela 3).

**Tabela 3:** Prevalência observada e esperada de combinações de fatores de risco associados ao estilo de vida em adultos quilombolas vivendo no município de Guanambi, Bahia, Brasil

Fatores de risco	Inatividade física	Ausência de consumo de frutas e vegetais	Consumo de bebida alcoólica	Tabagismo	Consumo de alimentos ricos em gordura e açúcar	O (%)	O/E	IC95%	OR bruto(IC95%)	OR ajustado (IC95%)
0	–	–	–	–	–	1,6	1,01*	0,78-1,25	0,81 (0,77-0,86)	–
1	+	–	–	–	–	0,3	0,61	0,39-0,84	0,82 (0,78-0,86)	–
1	–	+	–	–	–	0	0,00	–	–	–
1	–	–	+	–	–	14,2	1,01*	0,87-1,15	1,48 (0,62-3,50)	2,23 (0,69-7,17)
1	–	–	–	+	–	0	0,00	–	–	–
1	–	–	–	–	+	0,3	0,55	0,33-0,78	0,82 (0,78-0,86)	–
2	–	+	+	–	–	0	0,00	–	–	–
2	–	+	–	+	–	0	0,00	–	–	–
2	–	+	–	–	+	0	0,00	–	–	–
2	+	+	–	–	–	0	0,00	–	–	–
2	+	–	+	–	–	6,8	1,57*	1,35-1,79	0,56 (0,12-2,54)	0,51 (0,10-2,53)
2	+	–	–	+	–	0,3	0,67	0,45-0,89	0,82 (0,78-0,86)	–
2	+	–	–	–	+	0	0,00	–	–	–
2	–	–	+	+	–	10,3	0,80	0,64-0,95	1,07 (0,38-2,98)	1,28 (0,40-4,05)
2	–	–	+	–	+	2,3	0,48	0,26-0,69	0,75 (0,08-6,41)	1,01 (0,10-9,9)
2	–	–	–	+	+	1,6	3,21*	2,98-3,43	3,18 (0,52-19,52)	4,86 (0,63-37,3)
1	+	+	+	–	–	0	0,00	–	–	–
1	+	+	–	+	–	0	0,00	–	–	–
1	+	+	–	–	+	0	0,00	–	–	–
1	+	–	+	+	–	3,4	0,85	0,63-1,08	1,25 (0,25-6,09)	1,43 (0,26-7,68)
1	+	–	+	–	+	0	0,00	–	–	–
1	+	–	–	+	+	0,3	1,95*	1,79-2,10	0,82 (0,78-0,86)	–
1	–	+	+	+	–	0	0,00	–	–	–
1	–	+	+	–	+	0	0,00	–	–	–
1	–	+	–	+	+	0	0,00	–	–	–
1	–	–	+	+	+	6,8	1,53*	1,31-1,75	0,79 (0,75-0,84)	–
4	+	+	+	+	–	0	0,00	–	–	–
4	+	+	+	–	+	0	0,00	–	–	–
4	+	+	–	+	+	0	0,00	–	–	–
4	+	–	+	+	+	0,6	0,44	0,20-0,67	4,51 (0,27-73,3)	5,44 (0,26-111,5)
4	–	+	+	+	+	0	0,00	–	–	–
5	+	+	+	+	+	0	0,00	–	–	–

+ = presença de comportamentos não saudáveis;

– = ausência de comportamentos não saudáveis.

O/E: % Observado /% Esperado; IC: Intervalo de Confiança; OR: odds ratio; o modelo foi ajustado quanto a idade, estado civil, escolaridade e ocupação;

* estatisticamente significativo

A avaliação da associação entre todas as combinações de fatores de risco e a presença de diabesidade revelou associações da diabesidade com ausência de fatores de risco (OR=0,81; IC95% 0,77-0,86), e com inatividade física na ausência de outros fatores (OR=0,82; IC95% 0,78-0,86) somente na análise bruta (não ajustada) (Tabela 3).

## Discussão

Os resultados do presente estudo mostraram uma prevalência de diabesidade de aproximadamente 18,0%. Os escores de agrupamento mais altos foram observados para as combinações de consumo de bebida alcoólica na ausência de outros fatores de risco, seguido de consumo regular de bebida alcoólica e tabagismo, e consumo regular de bebida alcoólica, tabagismo e consumo de alimentos ricos em açúcar e gordura. Além disso, a ausência de todos os comportamentos de risco foi associada a uma prevalência mais baixa de diabesidade somente na análise não ajustada.

A prevalência de diabesidade observada aqui foi mais alta que a encontrada em um estudo conduzido em trabalhadores espanhóis (10,0%).^30^ Embora a diabesidade ter sido reconhecida por um tempo, poucos estudos avaliaram sua ocorrência em grupos populacionais específicos. Por outro lado, estudos conduzidos no Brasil^31,32^ e em outros países^30,33^ têm investigado continuamente a ocorrência de diabetes tipo 2 e obesidade.

Na população negra, estudos brasileiros relataram uma prevalência de diabetes e obesidade variando entre 9,8%^34^ e 23,5%^35^ e entre 27,7%^26^ e 56,6%,^32^ respectivamente.

Entre as combinações de comportamento avaliadas, a presença de consumo regular de bebida alcoólica entre as combinações com os escores de agrupamento mais altos chamou atenção. Ainda, as combinações de consumo regular de bebida alcoólica com tabagismo e consumo de alimentos ricos em açúcar e gordura foram prevalentes em adultos quilombolas.

Nossos resultados estão de acordo com os resultados relatados de Cardoso, Melo e César^36^ que demonstraram associações entre consumo de álcool e tabagismo em adultos quilombolas. Vale mencionar que o tabagismo aumenta o risco de se consumir bebidas alcoólicas^36^ e é um fator de risco importante para doenças crônicas não degenerativas.^37,38^ Esses achados indicam a falta de acesso a atividades de promoção da saúde, principalmente em populações vivendo em condições socioeconômicas desfavoráveis.

A análise dos dados do *2013 National Health Survey* (PNS) envolvendo populações urbanas mostrou que o consumo abusivo de álcool está associado com diabetes mellitus.^6^ Em um estudo avaliando quilombolas adultos, Campagna et al.^39^ demonstraram que ser um ex-tabagista tem um impacto negativo sobre o peso corporal e o controle glicêmico e consequentemente aumenta o risco de diabetes.

Em relação aos padrões de consumo alimentar no Brasil, um estudo prévio conduzido por Levy-Costa et al.^40^ mostrou um declínio no consumo de alimentos básicos e tradicionais, e um aumento no consumo de alimentos industrializados e com alto teor de gordura. Estudos com comunidades quilombola relataram um baixo consumo de frutas e vegetais nessas populações, o que contribui ao ganho de peso e a um aumento no risco cardiovascular.^41,42^

Queiroz et al.,^32^ que avaliaram adultos quilombolas na região de Minas Gerais, mostraram uma frequência mais alta de ingestão de alimentos com alto teor de açúcar (bolos, doces e biscoitos) durante uma semana em comparação a de frutas e verduras. Exposição à pobreza, uma condição comum em populações quilombolas, favorece o consumo de alimentos industrializados que são relativamente mais baratos, e menos nutritivos e mais ricos em energia.^43^ A condição econômica é um fator importante que influencia diretamente a vida das famílias quilombolas. Pelo fato de a renda familiar não ser suficiente, essas famílias não conseguem comprar alimentos altamente nutritivos, o que exerce uma influência negativa na saúde desses indivíduos.^44^

Em nosso estudo, a ausência de comportamentos de risco foi associada à presença de diabesidade somente na análise não ajustada. Esse resultado está, de certa forma, de acordo com os resultados de um estudo internacional conduzido com trabalhadores espanhóis que demonstrou uma relação da prevalência de diabesidade com a exposição a uma dieta não saudável ao coração e falta de participação em programas de exercício.^30^

Assim, em um contexto multifatorial, os mecanismos fisiológicos do envelhecimento, como um maior acúmulo de gordura abdominal, em conjunto com comportamentos de risco – que contribuem para um aumento nas citocinas pró-inflamatórias e uma redução nos níveis de lipoproteína de alta densidade – estão intimamente relacionados à diabesidade nessa população.^45,46^

Em relação à atividade física, Soares e Barreto^42^ relataram uma relação negativa entre obesidade e um nível de atividade física em adultos quilombolas. Pitanga et al.,^47^ que avaliaram adultos negros com idade entre 20 e 96 anos que viviam na cidade de Salvador, Bahia, demonstraram a existência de uma associação entre atividade física e diabetes mellitus.

A prevalência de inatividade física na população quilombola estudada (23,6%) é similar à encontrada em quilombolas no município de Vitória da Conquista, Bahia (26,3%).^41^ Atividade física regular está diretamente associada à melhoria e/ou manutenção da saúde em indivíduos de todas as idades, e está inversamente associada com diferentes fatores de risco à saúde.^48^

Apesar de a informação sobre a associação dos comportamentos de risco com a diabesidade ser incipiente, já está bem estabelecido na literatura que alimentação inadequada, consumo de bebida alcoólica, tabagismo, e falta de atividade física regular estão diretamente relacionados ao aumento na obesidade e a alterações nos índices glicêmicos. Ainda, esses hábitos possivelmente contribuem para o início e o agravamento de diabete mellitus tipo 2.^34,42^

De acordo com a literatura, o manejo da diabesidade deve consistir em estratégias terapêuticas combinadas, uma vez que tanto o diabetes como a obesidade requerem ações que estimulam mudanças no estilo de vida.^49^ Portanto, profissionais da saúde devem delinear uma abordagem multiprofissional para pacientes diabéticos e obesos que aborde mudanças dietéticas de um estilo de vida ativo.

Entre as limitações deste estudo, citamos o fato de que algumas variáveis foram autorrelatadas, o que pode causar possível viés de memória. Outra limitação é seu delineamento transversal, o que dificulta a determinação de uma relação causal entre os fatores estudados. Os pontos fortes deste estudo são a inclusão de uma amostra representativa de quilombolas de meia idade e idosos, comunidade que são ainda pouco investigadas, e o uso de instrumentos de avaliação previamente validados. Além disso, o estudo aborda um problema relevante, principalmente nessa população que é caracterizada por baixo nível educacional, baixa renda, e acesso insuficiente a serviços de saúde.

Finalmente, os resultados do presente estudo mostraram que o consumo de álcool foi o fator mais prevalente entre as principais combinações avaliadas. A presença de inatividade física, na ausência de outros fatores, foi associada à diabesidade somente na análise não ajustada.

## Conclusão

No geral, nossos achados reforçam a importância de se obter dados que auxiliarão na intervenção precoce a fim de se prevenir e controlar o ganho de peso e o diabetes, juntamente com investimentos em programas de promoção da saúde, tais como intervenções que encorajem alimentação saudável e atividade física, e restrinjam tabagismo e ingestão de álcool.
