# Supplementary material for: Cluster de Inatividade Física e Outros Fatores de Risco na Diabesidade em Adultos Quilombolas
Source: Arq Bras Cardiol. 2024 Nov 13;121(11):e20230715. [Article in Portuguese] doi: 10.36660/abc.20230715 (PMC11634302; doi:10.36660/abc.20230715)
Supplement: Translation

# Translation: Cluster of Physical Inactivity and Other Risk Factors and Diabesity in Quilombol Adults

**DOI:** 10.36660/abc.20230715i

**Authors:** Poliana Pereira Santana, Clarice Alves dos Santos, Ricardo Franklin de Freitas Mussi, Hector Luiz Rodrigues Munaro, Saulo Vasconcelos Rocha

**Affiliations:** 1 Jequié BA Brazil Universidade Estadual do Sudoeste da Bahia, Programa de Pós-Graduação em Educação Física UESB/UESC, Jequié, BA – Brazil; 1 Jequié BA Brazil Universidade Estadual do Sudoeste da Bahia, Departamento de Ciências Biológicas, Jequié, BA – Brazil; 3 Caitité BA Brazil Universidade do Estado da Bahia, Programa de Pós-Graduação em Ensino, Linguagem e Sociedade, Caitité, BA – Brazil; 4 Jequié BA Brazil Universidade Estadual do Sudoeste da Bahia, Departamento de Saúde II, Jequié, BA – Brazil; 5 Jequié BA Brazil Universidade Estadual do Sudoeste da Bahia, Departamento de Saúde, Jequié, BA – Brazil; 6 Feira de Santana BA Brazil Universidade Estadual de Feira de Santana, Programa de Pós-Graduação em Saúde Coletiva, Feira de Santana, BA – Brazil

**Keywords:** Risk-Taking, Diabetes Mellitus, Obesity, Quilombola Communities

## Abstract

**Background:**

Diabesity is a condition characterized by the coexistence of type 02 diabetes and obesity. The causes are multifactorial, resulting from a complex interaction of genetic and behavioral factors. Among the behavioral factors, there are physical inactivity, inadequate eating habits and excessive consumption of alcohol and tobacco.

**Objective:**

To investigate the clustering of physical inactivity and other risk factors and the association between risk factor combinations and the presence of diabesity in quilombola adults.

**Methods:**

Cross-sectional study involving a sample of 332 middle-aged and older adults (≥ 50 years) selected among participants in the "Epidemiological Profile of Quilombolas in Bahia" study. Data were collected by interview and anthropometric assessment. Descriptive statistics, cluster analysis, and multinomial logistic regression procedures were used for data analysis.

**Results:**

The highest prevalence of clustering was identified for the combinations of regular alcohol consumption in the absence of the other factors (O/E=14.2; 95%CI 0.87-1.15), followed by regular alcohol and tobacco consumption (O/E=10.3; 95%CI 0.64-0.95) and regular consumption of alcohol, tobacco and foods high in sugar and fat (O/E=6.8; 95%CI= 1.31-1.75). Unadjusted analysis revealed an association between physical inactivity in the absence of the other factors (OR=0.82; 95%CI 0.78-0.86) and diabesity.

**Conclusion:**

Alcohol consumption was the most prevalent factor among the largest combinations evaluated. Furthermore, the presence of physical inactivity without the other behaviors analyzed and the absence of all behaviors were associated with diabesity only in unadjusted analysis.

## Introduction

Diabesity is a term used to describe the adverse health effects of the simultaneous presence of two conditions: obesity and type 2 diabetes mellitus.^1^

Obesity is a prominent risk factor for type 2 diabetes since it triggers insulin resistance and metabolic complications.^2,3^ It is estimated that between 80 and 90% of individuals with type 2 diabetes are obese and the risk is directly linked to the increase in body mass index.^4^ Projections indicate that, by 2025, more than 300 million people will have obesity-associated type 2 diabetes mellitus.^5^

The causes of obesity and diabetes are multifactorial and are the result of a complex interaction between genetic and behavioral factors. Important behavioral factors include inadequate eating habits, excessive consumption of alcohol and tobacco, and physical inactivity.^6,7^

Physical inactivity is a serious public health problem and is responsible for more than three million deaths per year worldwide. Despite this, estimates indicate that one third of the world population older than 15 years does not meet the minimum recommendation of the World Health Organization (WHO), *i.e*., performing at least 150 minutes of physical activity per week.^8^

In addition to physical inactivity, inadequate eating habits seem to directly influence the development of obesity and type 2 diabetes mellitus. The increased consumption of processed carbohydrates with a high glycemic index can lead to hormonal changes that promote the accumulation of adipose tissue, exacerbate hunger, and reduce energy expenditure.^9^ On the other hand, the WHO recommends the consumption of fruits and vegetables in order to reduce the incidence of cardiovascular diseases and certain types of cancer, as well as to prevent and treat overweight and diabetes.^10^

Another risk behavior that deserves attention is tobacco consumption. Evidence indicates a significant association between smoking and a higher prevalence of overweight and obesity, as the metabolic effects of nicotine favor the accumulation of abdominal fat.^11^ Furthermore, smoking also promotes important metabolic changes involved in the development of type 2 diabetes. Nicotine has been shown to reduce the secretion of insulin, to increase plasma cortisol levels, and to induce cell dysfunction and apoptosis.^12^

Regarding abusive alcohol consumption, studies have shown that type 2 diabetes is caused by a direct action of alcohol^13^ or by other unhealthy behaviors observed in alcohol drinkers.^6^

According to the literature, the health-disease process is the result of the individuals’ living conditions, *i.e*., their habits and lifestyle, socioeconomic conditions, culture, and the environment where they live.^14^ From this perspective, it is evident that unfavorable socioeconomic conditions increase the susceptibility to risk behaviors^15^ and to the development of chronic diseases.^16^

Within this context, Brazilian studies on social inequalities that address race/skin color have demonstrated poor health outcomes in the black population.^17,18^

Among the different Brazilian black population profiles, the quilombola population faces a situation of social vulnerability,^18^ because of their difficult access to public policies and inadequate socioeconomic conditions.^19,20^

Furthermore, diabetes is known to affect black populations differently, influenced by genetic, environmental and behavioral factors, as well as by socioeconomic vulnerability.^21,22^ A similar scenario seems to apply to obesity.

Therefore, economic conditions can negatively influence the quality of life of adult and elderly quilombolas, who experience conditions that are unfavorable to healthy aging.^23^ Previous studies have investigated risk behaviors and the individual conditions of diabetes^6^ and obesity^24^ in adults and older adults. The results indicated that these conditions tend to occur simultaneously, a fact that has important short-, medium- and long-term health implications. However, studies analyzing combinations of risk factors related to diabesity are still incipient, especially in elderly quilombolas.

Within this context, the aim of the present study was to investigate physical inactivity and other risk factors, and the association between risk factor combinations and the presence of diabesity in adult quilombolas.

## Methods

A population-based, cross-sectional, descriptive study involving a population of quilombo remnants from the microregion of Guanambi, Bahia, Brazil, was conducted from April to November 2016. The region is composed of 18 municipalities and had 42 certified quilombos during the sampling period, distributed across 10 of these municipalities.^25^

The sample size was calculated assuming a prevalence of unknown outcome of 50%, confidence interval of 95%, sampling error of 5%, and an effect size of 1.5 for a one-stage cluster sample; 30% for refusals and 20% for losses and confounding were added. Further details regarding the sample selection process have been published previously.^26^ For the present study, data of middle-aged and older adults (≥ 50 years) were included, and the sample consisted of 348 individuals (40.8% of the total population).

Data were collected by interview and anthropometric measurements, which were conducted by teams of healthcare workers and/or students according to their qualifications, after they had been trained in their respective function. The following data were collected: sex (male or female); age in complete years and categorized by age group (50 to 74 or ≥ 75 years); occupation (unpaid or paid work); marital status (with or without a partner); educational level (≤ 5 or > 5 years of schooling); tobacco consumption (yes: smokes or has smoked; no: never smoked); regular alcohol consumption (yes: drinks or sporadically drinks, no: never drinks); regular consumption of fruits and vegetables (yes: always, almost always, sometimes, no: never); consumption of foods high in sugar and fat (yes: always, almost always, sometimes, no: never); presence of diabetes (yes or no).

Physical activity was evaluated using the short version of the International Physical Activity Questionnaire (IPAQ),^27^ which assesses the weekly time spent in moderate or vigorous physical activity across different life domains (work, household tasks, transportation, and leisure). Subjects whose sum of moderate or vigorous physical activity in the different domains was less than 150 minutes per week were classified as insufficiently active.

Diabesity was classified based on the self-reported diagnosis of diabetes mellitus and the measurement of waist circumference.^28^ The presence of diabesity was defined when participants with a self-reported diagnosis of diabetes simultaneously had a waist circumference > 90 cm for men and > 80 cm for women.^29^

Descriptive statistics (simple and relative frequencies and measures of dispersion) were used for univariate analysis of the data. For bivariate analysis, Pearson's chi-square test was applied to compare the variables between men and women using the Statistical Package for the Social Sciences (SPSS version 22 for Windows).

The simultaneous presence or clustering of factors related to diabesity was analyzed by calculating the joint probability of the exhibited behaviors. The presence of clustering was evaluated by comparing the observed (O) and expected (E) prevalence. Clustering is defined when the O/E ratio > 1.0. For analysis of the association of the predictors with diabesity, the odds ratio (OR) was estimated from the binary logistic regression model.

The "Epidemiological Profile of Quilombolas in Bahia" study was approved by the Ethics Committee of Universidade Estadual do Sudoeste da Bahia (Opinion No. 1.386.019/2016) and was conducted following the Brazilian guidelines on research involving humans, in accordance with Resolution 466/2012 of the National Health Council.

## Results

The mean age of the individuals included in the study was 61.4 ± 9.5 years and most of them were female (52.5%). The prevalence of physical inactivity, lack of fruit and vegetable consumption, alcohol consumption, smoking and consumption of high-sugar and high-fat foods was 23.6%, 41.8%, 25.6%, 47.9% and 89.9%, respectively. Furthermore, 17.8% of the respondents had diabesity (Table 1).

**Table 1:** Association between sociodemographic characteristics and lifestyle-related risk factors in adult quilombolas living in the municipality of Guanambi, Bahia, Brazil

Variables		Total sample		Physical inactivity		Lack of fruit and vegetable consumption		Alcohol consumption		Tobacco consumption		Consumption of high-sugar/high-fat foods	
		n	%	n	%	n	%	n	%	n	%	n	%
**Total**		320		61	23.6	132	41.8	82	25.6	148	47.9	284	89.9
**Sex**				p=0.59		p=0.26		p=0.00		p=0.00		p=0.03	
	Male	153	47.8	31	20.3	68	44.4	68	44.4	103	67.3	130	84.9
	Female	167	52.5	30	17.9	64	38.3	14	8.4	45	26.9	154	92.2
**Age group (years)**				p=0.38		p=0.39		p=0.70		p=0.26		p=0.52	
	50 to 74	277	86.6	52	18.7	117	42.2	72	26.0	124	44.8	246	88.8
	75 or more	43	13.4	9	20.9	15	34.9	10	23.2	24	55.8	38	88.4
**Occupation**				p=0.32		p=0.29		p=0.08		p=0.33		p=0.16	
	Paid work	274	85.6	51	18.6	116	42.3	75	27.4	129	47.1	240	87.5
	Unpaid work	46	14.4	10	21.7	16	34.7	7	15.2	19	41.3	44	95.6
**Marital status**				p=0.65		p=0.54		p=0.61		p=0.33		p=0.36	
	With a partner	259	80.9	48	18.5	109	42.1	69	26.6	124	47.8	232	89.6
	Without a partner	60	18.8	13	21.6	23	38.3	13	21.6	24	40.0	52	86.6
**Educational level**				p=0.001		p=0.07		p=0.66		p=0.86		p=0.71	
	≤ 5 years	242	75.6	37	15.3	108	44.6	65	26.8	113	46.7	213	88.0
	> 5 years	47	14.7	13	27.6	15	31.9	10	21.3	22	46.8	43	91.5
**Diabesity**				p=0.46		p=0.72		p=0.12		p=0.91		p=0.70	
	Yes	57	82.2	12	21.0	25	43.8	10	17.5	26	45.6	52	91.3
	No	263	17.8	31	54.3	32	56.2	47	82.5	29	50.9	5	8.7

The results of Table 2 show the lack of associations between individual risk factors and the presence of diabesity.

**Table 2:** Association between each lifestyle-related risk factors and the presence of diabesity

Risk factor Crude	OR (95%CI)	Adjusted OR (95%CI)
Lack of fruit and vegetable consumption	1.10 (0.62-1.98)	0.80 (0.40-1.59)
Regular alcohol consumption	1.21 (0.44-3.29)	0.90 (0.28-2.89)
Tobacco consumption	0.97 (0.54-1.73)	0.72 (0.34-1.55)
Consumption of high-sugar foods	0.56 (0.27-1.17)	1.32 (0.55-3.20)
Physical inactivity	1.31 (0.63-2.76)	0.60 (0.27-1.31)

Adjusted for sex, age, educational level, marital status, and occupation. OR: odds ratio; CI: confidence interval.

Among the 32 possible combinations, cluster scores were obtained for six combinations. The highest scores were identified for the combinations of regular alcohol consumption in the absence of the other factors (O/E=14.2), followed by regular alcohol and tobacco consumption (O/E=10.3), and regular consumption of alcohol, tobacco and high-sugar and high-fat foods (O/E=6.8) (Table 3).

**Table 3:** Observed and expected prevalence of the combinations of lifestyle-related risk factors in adult quilombolas living in the municipality of Guanambi, Bahia, Brazil

Risk factors	Physical inactivity	Lack of fruit and vegetable consumption	Alcohol consumption	Tobacco consumption	Consumption of high-sugar/ high-fat foods	O (%)	O/E	95%CI	Crude OR (95%CI)	Adjusted OR (95%CI)
0	–	–	–	–	–	1.6	1.01*	0.78-1.25	0.81 (0.77-0.86)	–
1	+	–	–	–	–	0.3	0.61	0.39-0.84	0.82 (0.78-0.86)	–
1	–	+	–	–	–	0	0.00	–	–	–
1	–	–	+	–	–	14.2	1.01*	0.87-1.15	1.48 (0.62-3.50)	2.23 (0.69-7.17)
1	–	–	–	+	–	0	0.00	–	–	–
1	–	–	–	–	+	0.3	0.55	0.33-0.78	0.82 (0.78-0.86)	–
2	–	+	+	–	–	0	0.00	–	–	–
2	–	+	–	+	–	0	0.00	–	–	–
2	–	+	–	–	+	0	0.00	–	–	–
2	+	+	–	–	–	0	0.00	–	–	–
2	+	–	+	–	–	6.8	1.57*	1.35-1.79	0.56 (0.12-2.54)	0.51 (0.10-2.53)
2	+	–	–	+	–	0.3	0.67	0.45-0.89	0.82 (0.78-0.86)	–
2	+	–	–	–	+	0	0.00	–	–	–
2	–	–	+	+	–	10.3	0.80	0.64-0.95	1.07 (0.38-2.98)	1.28 (0.40-4.05)
2	–	–	+	–	+	2.3	0.48	0.26-0.69	0.75 (0.08-6.41)	1.01 (0.10-9.9)
2	–	–	–	+	+	1.6	3.21*	2.98-3.43	3.18 (0.52-19.52)	4.86 (0.63-37.3)
3	+	+	+	–	–	0	0.00	–	–	–
3	+	+	–	+	–	0	0.00	–	–	–
3	+	+	–	–	+	0	0.00	–	–	–
3	+	–	+	+	–	3.4	0.85	0.63-1.08	1.25 (0.25-6.09)	1.43 (0.26-7.68)
3	+	–	+	–	+	0	0.00	–	–	–
3	+	–	–	+	+	0.3	1.95*	1.79-2.10	0.82 (0.78-0.86)	–
3	–	+	+	+	–	0	0.00	–	–	–
3	–	+	+	–	+	0	0.00	–	–	–
3	–	+	–	+	+	0	0.00	–	–	–
3	–	–	+	+	+	6.8	1.53*	1.31-1.75	0.79 (0.75-0.84)	–
4	+	+	+	+	–	0	0.00	–	–	–
4	+	+	+	–	+	0	0.00	–	–	–
4	+	+	–	+	+	0	0.00	–	–	–
4	+	–	+	+	+	0.6	0.44	0.20-0.67	4.51 (0.27-73.3)	5.44 (0.26-111.5)
4	–	+	+	+	+	0	0.00	–	–	–
5	+	+	+	+	+	0	0.00	–	–	–

+ = presence of unhealthy behaviors;

– = absence of unhealthy behaviors.

O/E: % observed/% expected; CI: confidence interval; OR: odds ratio. The model was adjusted for age, marital status, educational level, and occupation.

* Statistically significant.

Evaluation of the association between all combinations of risk factors and the presence of diabesity revealed associations of diabesity with the absence of risk factors (OR=0.81; 95%CI 0.77-0.86) and with physical inactivity in the absence of the other factors (OR=0.82; 95%CI 0.78-0.86) only in the unadjusted analysis (Table 3).

## Discussion

The results of the present study showed a prevalence of diabesity of approximately 18.0%. The highest cluster scores were observed for the combinations of alcohol consumption in the absence of other risk factors, followed by regular consumption of alcohol and tobacco, and regular consumption of alcohol, tobacco and high-sugar and high-fat foods. Furthermore, the absence of all risk behaviors was associated with a lower prevalence of diabesity only in the unadjusted analysis.

The prevalence of diabesity observed here was higher than that found in a study conducted on Spanish workers (10.0%).^30^ Although diabesity has been recognized for some time, few studies have evaluated its occurrence in specific population groups. On the other hand, studies conducted in Brazil^31,32^ and in other countries^30,33^ have continuously investigated the occurrence of type 2 diabetes mellitus and obesity.

In the black population, Brazilian studies have reported a prevalence of diabetes and obesity ranging from 9,8%^34^ to 23,5%^35^ and from 27,7%^26^ to 56,6%,^32^ respectively.

Among the behavior combinations evaluated, the presence of regular alcohol consumption among those with the highest cluster scores called attention. Moreover, the combinations of regular alcohol consumption with the consumption of tobacco and high-sugar/high-fat foods were prevalent among adult quilombolas.

Our results are in line with the findings of Cardoso, Melo and César^36^ who demonstrated associations between alcohol consumption and smoking in adult quilombola populations. It is worth mentioning that the continuous use of tobacco increases the risk of consuming alcoholic beverages^36^ and is a leading risk factor for non-communicable chronic degenerative diseases.^37,38^ These findings indicate the lack of access to health promotion activities, especially among populations living under unfavorable socioeconomic conditions.

Analysis of the data from the 2013 National Health Survey (PNS) involving urban populations showed that abusive alcohol consumption is associated with diabetes mellitus.^6^ In a study evaluating adult quilombolas, Campagna et al.^39^ demonstrated that being a former smoker has a negative impact on body weight and glycemic control and consequently increases the risk of diabetes.

Regarding food consumption patterns in Brazil, a previous study conducted by Levy-Costa et al.^40^ showed a decline in the consumption of basic and traditional foods, while the consumption of industrialized and high-fat foods had increased. Studies on quilombola communities have reported a low consumption of fruits and vegetables in these populations, which contributes to weight gain and an increase in cardiovascular risk.^41,42^

Queiroz et al.,^32^ who evaluated adult quilombolas in the region of Minas Gerais, showed a higher frequency of the intake of sweet foods (cakes, candies, and cookies) over one week compared to fruit and vegetable consumption. Exposure to poverty, a common condition among quilombola populations, favors the consumption of industrialized foods that are less costly but have a low nutritional value and are high in energy.^43^ The economic condition is an important factor that directly influences the life of quilombola families. Because the household income is often not sufficient, these families are unable to purchase highly nutritious foods, which negatively influence the health status of these individuals.^44^

In our study, the absence of risk behaviors was associated with the presence of diabesity only in the unadjusted analysis. This result somehow agrees with the findings of an international study conducted with Spanish workers that demonstrated a relationship of the prevalence of diabesity with exposure to a non-heart-healthy diet and lack of participation in exercise programs.^30^

Thus, in a multifactorial context, physiological mechanisms of aging such as higher abdominal fat deposition, in conjunction with risk behaviors – which contribute to an increase in proinflammatory cytokines and a reduction in high-density lipoprotein levels – are closely related to diabesity in this population.^45,46^

With regard to physical activity, Soares and Barreto^42^ reported a negative relationship between obesity and the level of physical activity in adult quilombolas. Pitanga et al.,^47^ who evaluated black adults aged 20 to 96 years living in the city of Salvador, Bahia, demonstrated an association between physical activity and diabetes mellitus.

The prevalence of physical inactivity in the quilombola population studied (23.6%) is similar to the 26.3% found among quilombolas in the municipality of Vitória da Conquista, Bahia.^41^ Regular physical activity is directly linked to health improvement and/or maintenance in individuals of all ages and is inversely associated with different health risk factors.^48^

Despite the incipient information about the association of risk behaviors with diabesity, it is well established in the literature that inadequate eating habits, alcohol and tobacco consumption and lack of regular physical activity are directly related to the increase in obesity and changes in glycemic indexes, and possibly contribute to the onset and aggravation of type 2 diabetes mellitus.^34,42^

According to the literature, the management of diabesity should consist of combined treatment strategies since both diabetes and obesity require actions that encourage lifestyle changes.^49^ Thus, healthcare professionals should design a multidisciplinary approach for diabetic and obese patients that addresses dietary changes and an active lifestyle.

Among the limitations of this study, we cite the fact that some variables were self-reported, which can cause possible memory bias. Another limitation is its cross-sectional design, which impairs the determination of a causal relationship between the factors studied. The strengths of this study are the inclusion of a representative sample of middle-aged and elderly quilombolas, communities that are still little investigated, and the use of previously validated assessment instruments. In addition, the study addresses a relevant problem, especially in this population that is characterized by a low educational level, low income, and insufficient access to health services.

Finally, the results of the present study showed that alcohol consumption was the most prevalent factor among the main combinations evaluated. The presence of physical inactivity, in the absence of the other factors, was associated with diabesity only in the unadjusted analysis.

## Conclusion

Taken together, our findings reinforce the importance of obtaining data that will assist in early intervention in order to prevent and control weight gain and diabetes, together with investments in health promotion programs such as interventions that encourage healthy eating, physical activity, and restrict tobacco and alcohol.
